# Supplementary material for: E-cadherin integrates mechanotransduction and EGFR signaling to control junctional tissue polarization and tight junction positioning
Source: Nat Commun. 2017 Nov 1;8:1250. doi: 10.1038/s41467-017-01170-7 (PMC5665913; doi:10.1038/s41467-017-01170-7)
Supplement: Supplementary file 1 — Supplementary Information [file 41467_2017_1170_MOESM1_ESM.pdf]

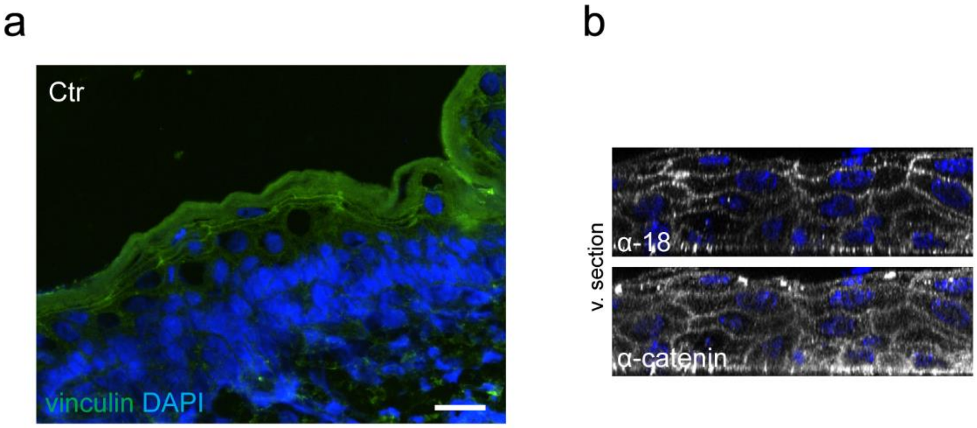

Supplementary Figure 1 Mechanosensitive AJ in the stratum granulosum 2 of mouse epidermis. (a) Staining for vinculin in newborn mouse epidermis (conventional sections). Scale bar, 20µm. (b) Staining for tension-sensitive epitope (α18) and total α-catenin showing that mechanosensitive α-catenin is only present in SG2. Single channels corresponding to Fig. 1c.

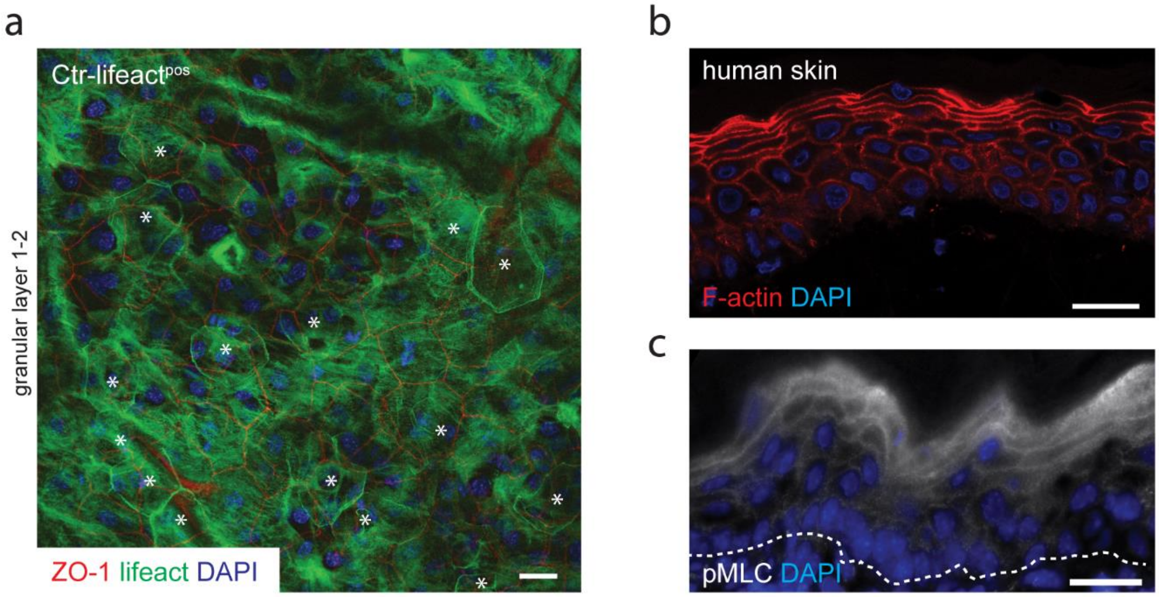

Supplementary Figure 2 F-actin shows basal to apical epidermal tissue polarization with scattered F-actin^{high} cells in the SG1 layer. (a) Partial confocal stack projection from a newborn mouse epidermal whole mount. Fluorescence of endogenously expressed lifeact-GFP is shown in green. F-actin high cells in the granular layer 1 layer are marked by asterisks. ZO-1 (red) visualizes TJs in the granular layer 2. (b) F-actin organization in normal human epidermis (conventional section). (c) Staining for active myosin (pSer20) in newborn mouse epidermis showing increased myosin activation in the granular layer (conventional sections). Scale bars, 20µm.

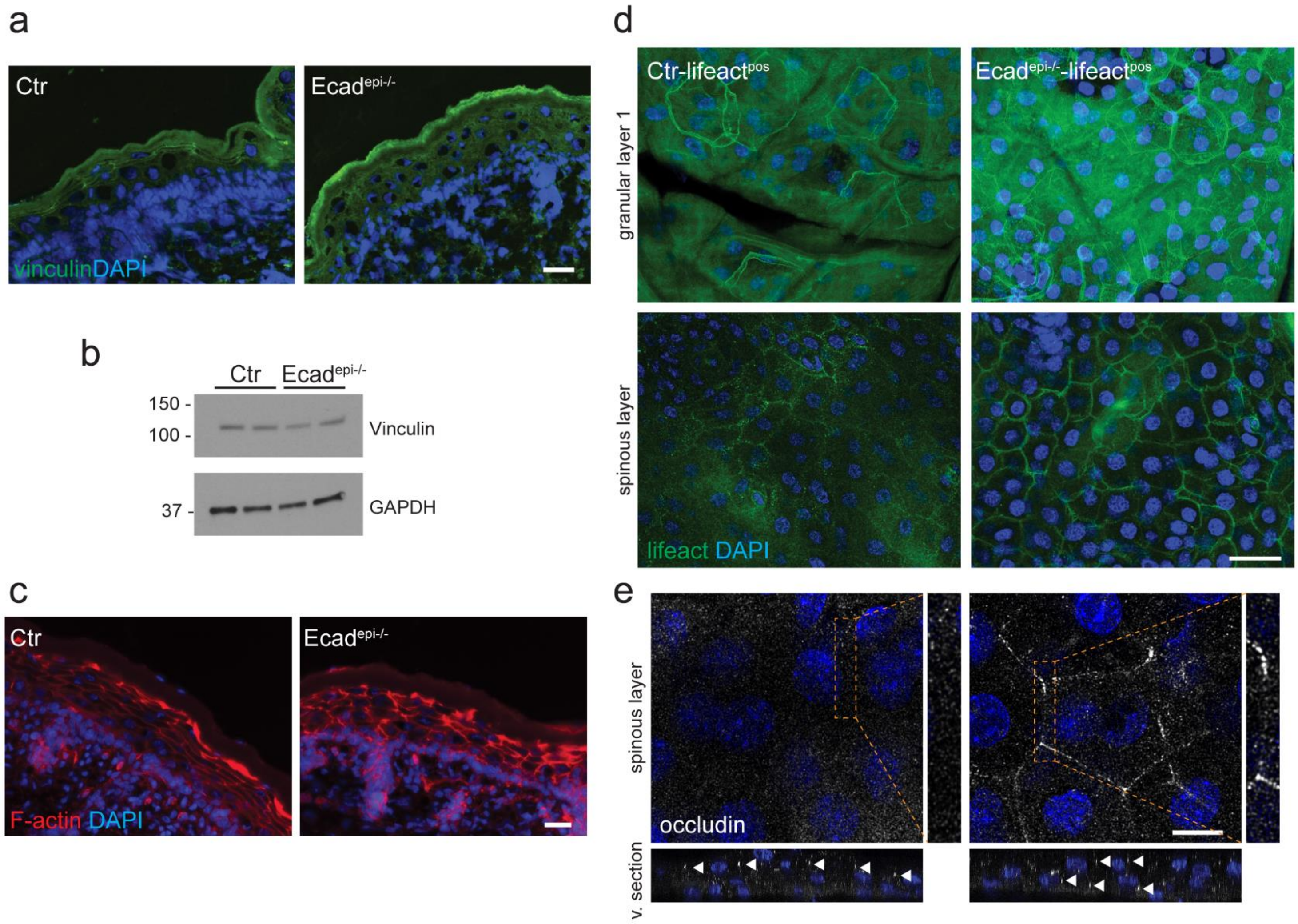

Supplementary Figure 3 E-cadherin regulates F-actin and cell-cell junction organization. (a) Staining for vinculin in newborn mouse epidermis showing loss of junctional vinculin in the granular layer 2. (b) Western blot analysis of vinculin total protein levels in mouse epidermis. (c) F-actin (Phalloidin) organization across epidermal layers showing depolarized F-actin organization upon loss of E-cadherin. (a,c) Conventional sections of Control (Ctr) and E-cadherin (ecad)$^{epi-/-}$ mice. Scale bars, 20µm. (d) Partial confocal stack projections from newborn mouse epidermal whole mounts of Life-act-GFP mice showing GFP in green to avoid potential staining artifacts by Phalloidin, DAPI was used to mark nuclei. Note that the localization of lifeact-GFP mirrors Phalloidin stainings shown in Fig. 2, 3. Scale bar, 30µm. (e) Partial confocal stack projections and virtual sections from newborn mouse epidermal whole mounts stained for occludin showing increased cortical concentration in the lower layers of E-cadherin deficient epidermis. Scale bar, 20µm.

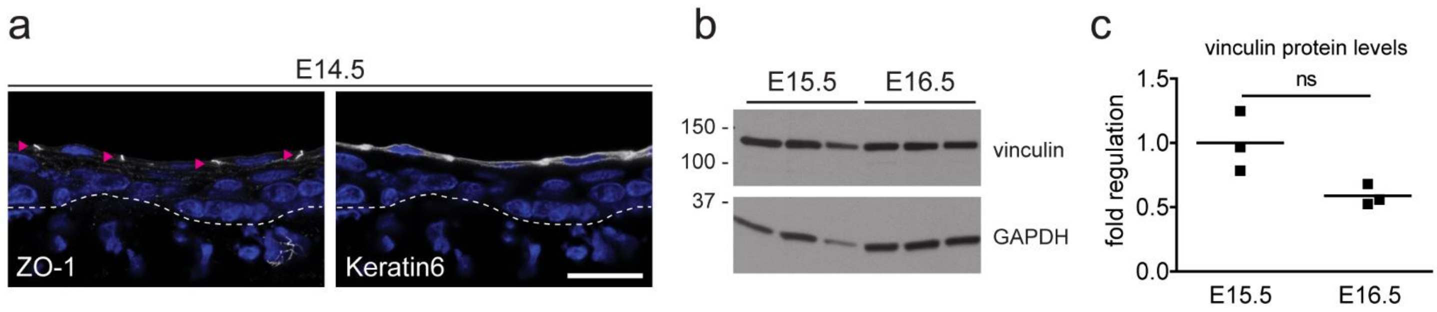

Supplementary Figure 4 Epidermal tight junction formation during mouse development. (a) Staining of E14.5 embryo skin for ZO-1 (tight junctions) and keratin-6 to mark periderm. Nuclei were counterstained with DAPI. Magenta arrowheads mark peridermal tight junctions. Scale bar, 20µm. (b,c) Western blot example and quantification of vinculin protein levels during embryonic epidermal barrier formation.

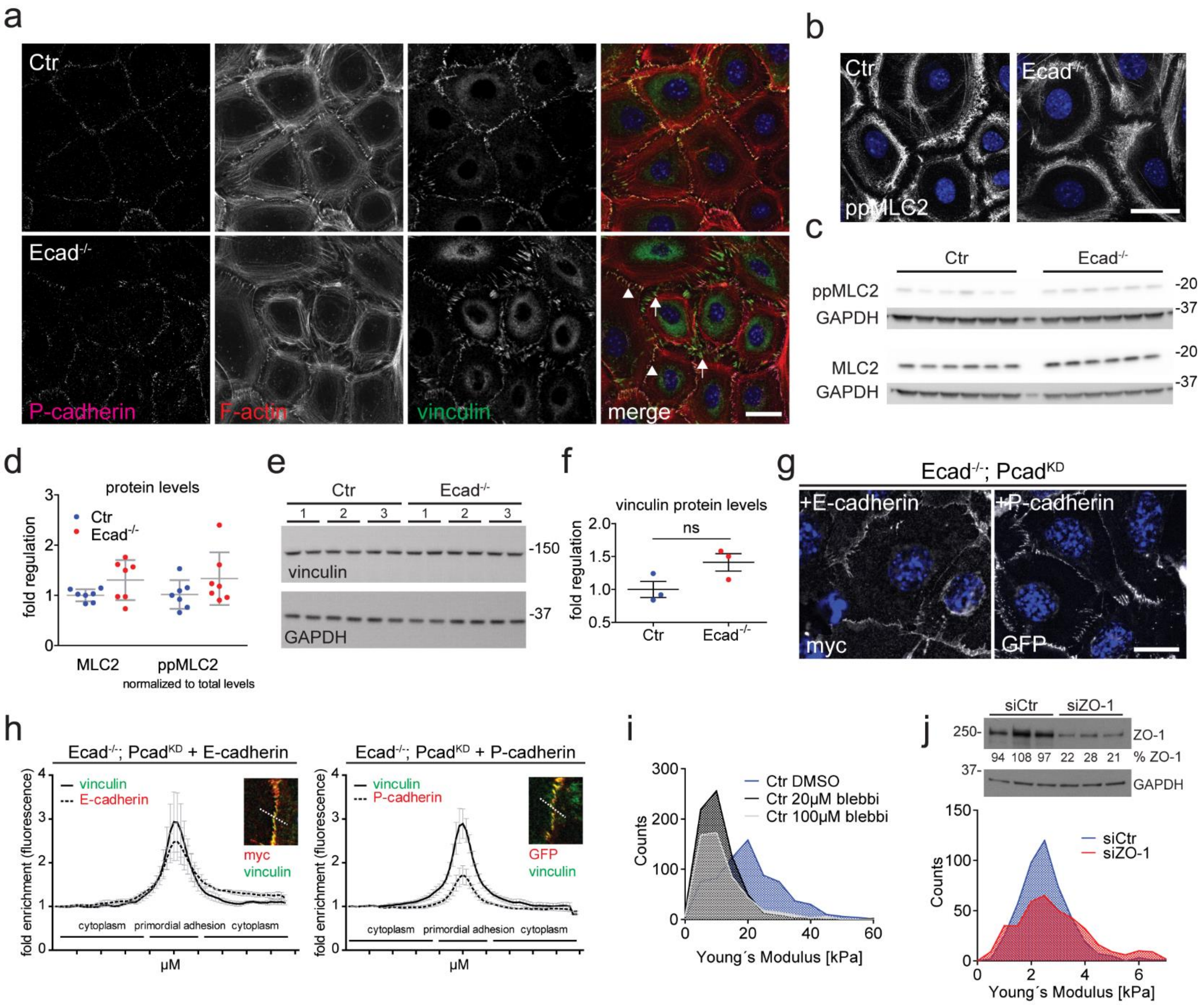
a
Ctr
Ecad-/-
P-cadherin
F-actin
vinculin
merge
b
Ctr
Ecad-/-
ppMLC2
c
Ctr
Ecad-/-
ppMLC2
GAPDH
MLC2
GAPDH
-20
-37
d
protein levels
Ctr
Ecad-/-
fold regulation
MLC2
ppMLC2
normalized to total levels
e
Ctr
Ecad-/-
1 2 3 1 2 3
vinculin
GAPDH
-150
-37
f
vinculin protein levels
ns
fold regulation
Ctr
Ecad-/-
g
Ecad-/-; PcadKD
+E-cadherin
+P-cadherin
myc
GFP
h
Ecad-/-; PcadKD + E-cadherin
Ecad-/-; PcadKD + P-cadherin
vinculin
E-cadherin
P-cadherin
myc
GFP
vinculin
fold enrichment (fluorescence)
cytoplasm
primordial adhesion
cytoplasm
µM
i
Ctr DMSO
Ctr 20µM blebbi
Ctr 100µM blebbi
Counts
Young´s Modulus [kPa]
j
siCtr
siZO-1
250-
ZO-1
94 108 97 22 28 21 % ZO-1
37-
GAPDH
siCtr
siZO-1
Counts
Young´s Modulus [kPa]

Supplementary Figure 5 Impaired junctional coupling but not contractility affects *in vitro* barrier function upon loss of E-cadherin. (a) Immunofluorescence analysis for P-cadherin, F-actin (phalloidin) and vinculin in Control (Ctr) and E-cadherin(Ecad)$^{-/-}$ keratinocytes cultured for 2h in high Ca^{2+}. (b) Immunofluorescence analysis of MLC2 activation (Thr18/Ser19) during primordial adhesion zipper formation (2h after Ca^{2+} switch). (c,d) Western blot example and quantification of total and phosphorylated MLC2 (Thr18/Ser19) levels in Ctr and Ecad$^{-/-}$ keratinocytes normalized to GAPDH. Means (lines) of n=7 biological replicates (dots). (e,f) Western blot example and quantification of vinculin in Ctr and Ecad$^{-/-}$ keratinocytes normalized to GAPDH. P=0.2; n=3 biological replicates with Mann-Whitney test. (g) Immunofluorescence analysis for transfected E-cadherin (myc) or P-cadherin (GFP) in Ecad$^{-/-}$;P-cadherin(Pcad)^{KD} keratinocytes showing that both rescue AJ zipper formation. Scale bar, 20µm. (h) Plot profile of vinculin recruitment to early AJ in Ecad$^{-/-}$;Pcad^{KD} cells transfected with either E-cadherin or P-cadherin. Mean plot profiles of n=3 independent experiments, 30 measurements per experiment. (a,b) Representative image of n≥3 biological replicates each. (i) Histogram showing the distribution of Young's moduli obtained from a representative indentation experiment from primary keratinocyte cell lines after 48h in high Ca^{2+} treated with indicated doses of Blebbistatin for 2h (n>500 measurements each dose). (j) Histogram showing the distribution of Young's moduli obtained from a representative indentation experiment from primary keratinocyte cell lines after 48h in high Ca^{2+} transfected with either Ctr or ZO-1 siRNA (siCtr: n=459, siZO-1: n=394 measurements). Western blot analysis for knockdown efficiency of ZO-1 is shown in the top. Scale bars, 20µm. (a,b,g) Nuclei were counterstained with Dapi (blue)

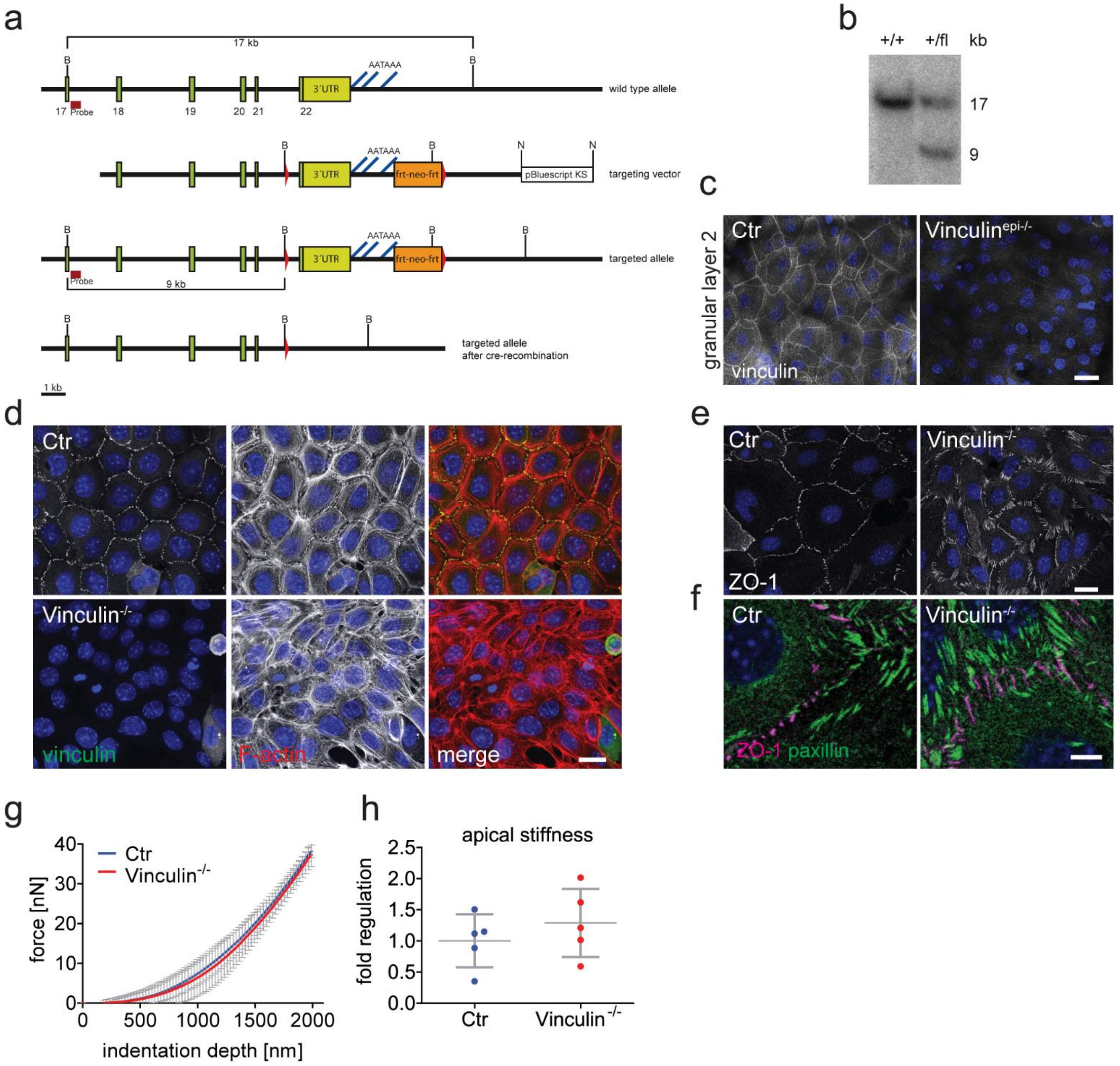

a
17 kb
B
AATAAA
3´UTR
wild type allele
17
Probe
18
19
20
21
22
targeting vector
frt-neo-frt
N
pBluescript KS
targeted allele
9 kb
targeted allele
after cre-recombination
1 kb
b
+/+
+/fl
kb
17
9
c
granular layer 2
Ctr
Vinculin$^{epi-/-}$
vinculin
d
Vinculin$^{-/-}$
vinculin
F-actin
merge
e
ZO-1
f
ZO-1 paxillin
g
force [nN]
indentation depth [nm]
h
apical stiffness
fold regulation

Supplementary Figure 6 Epidermal deletion of Vinculin affects de novo cell adhesion formation (a) Targeting strategy for the conditional vinculin knockout. The structure of the 3´end of the vinculin gene is shown at the top. Exons are indicated by green boxes. The targeting construct is shown in the center. LoxP sites (red triangles) were introduced upstream and downstream of mRNA polyadenylation signals (blue lines). A neomycin resistance cassette flanked by frt sites is located 1,8 kb downstream of the 3´UTR. The transgenic allele is shown at the bottom. B=BamHI; N=NotI. (b) Southern blot analysis. DNAs from G418 resistant ES cell clones were digested with BamHI and analyzed by southern blot analysis with the probe shown in panel (a). The wild-type allele is represented by a 17 kb band, the correctly targeted allele by a 9 kb band. (c) Immunofluorescence analysis for vinculin in the granular layer 2 (SG2) of Control (Ctr) and vinculin$^{epi-/-}$ epidermis. Deconvolved confocal stack projections from newborn mouse epidermal whole mounts. Scale bar, 20µm. (d) Immunofluorescence analysis for vinculin and F-actin (phalloidin) in Ctr and vinculin$^{-/-}$ keratinocytes 2h after switching to high Ca^{2+}, showing efficient deletion of vinculin and disorganization of F-actin in early adherens junctions. Scale bar, 20µm. (e,f) Staining for ZO-1 (e) and ZO-1/paxillin (f) after 2h in high Ca^{2+}. Nuclei were stained with DAPI (blue). Scale bar, 20µm (e), 5µm (f). (g) AFM force-indentation plots showing the average forces needed to reach a certain indentation depth. Mean plots of n=12 representative measurements including 3 primary Ctr and vinculin$^{-/-}$ cell lines are shown. (h) Quantification of the difference in Young's moduli in Ctr and vinculin$^{-/-}$ cells. n=5 biological replicates (>300 measurements per replicate).

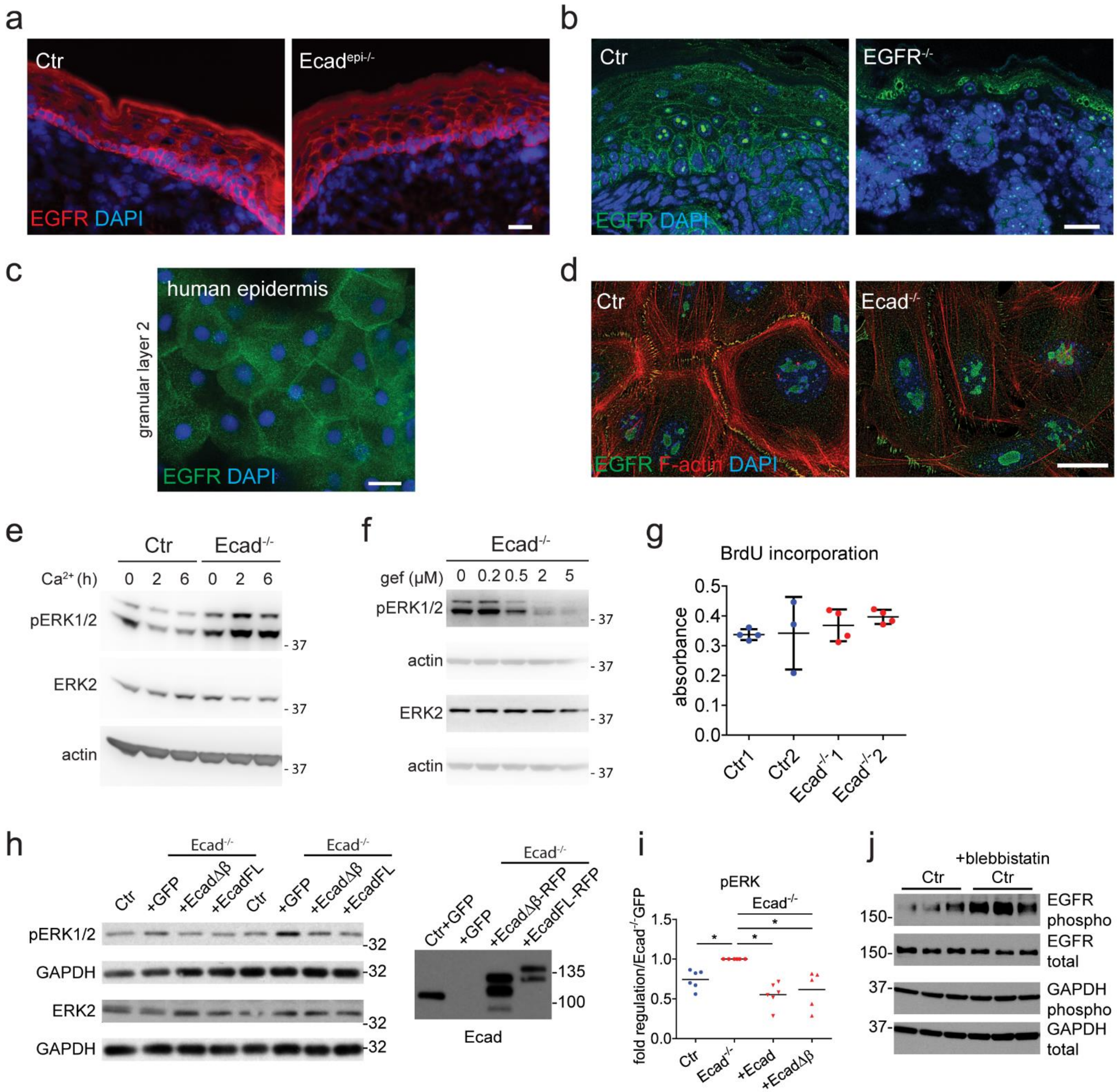
a
Ctr
EGFR DAPI
Ecadepi-/-
b
Ctr
EGFR DAPI
EGFR-/-
c
human epidermis
granular layer 2
EGFR DAPI
d
Ctr
EGFR F-actin DAPI
Ecad-/-
e
Ctr
Ecad-/-
Ca2+ (h)
0 2 6 0 2 6
pERK1/2
ERK2
actin
- 37
f
Ecad-/-
gef (μM)
0 0.2 0.5 2 5
pERK1/2
actin
ERK2
actin
- 37
g
BrdU incorporation
absorbance
Ctr1
Ctr2
Ecad-/-1
Ecad-/-2
h
Ecad-/-
Ctr
+GFP
+EcadΔβ
+EcadFL
pERK1/2
GAPDH
ERK2
GAPDH
-32
Ctr+GFP
+GFP
+EcadΔβ-RFP
+EcadFL-RFP
-135
-100
Ecad
i
pERK
Ecad-/-
fold regulation/Ecad-/-GFP
Ctr
Ecad-/-
+Ecad
+EcadΔβ
j
+blebbistatin
Ctr
EGFR phospho
EGFR total
GAPDH phospho
GAPDH total
150-
37-

Supplementary Figure 7 E-cadherin regulates EGFR localization in mouse and human epidermis. (a) Immunohistochemical analysis for EGFR in Ctr and Ecad$^{epi-/-}$ newborn epidermis to confirm EGFR localization using a different antibody (Millipore). (b) Immunohistochemical analysis for EGFR in the epidermis of Control (Ctr) and EGFR$^{-/-}$ mice to test specificity of EGFR antibody used for immunolocalization of EGFR in epidermal whole mounts. (a,b) conventional section. (c) Staining for EGFR in adult human epidermis, granular layer 2. Partial confocal stack projection of a human epidermal whole mount. (d) Immunofluorescence analysis of EGFR recruitment to E-cadherin dependent cell-cell contacts in keratinocytes 2h after switching to high Ca^{2+} (deconvolved). (e) Western blot analysis for indicated proteins in Ctr and Ecad$^{-/-}$ keratinocytes at indicated time points after switching to high calcium. (f) Western blot analysis of indicated proteins in Ecad$^{-/-}$ keratinocytes cultured in high calcium and treated with different concentrations of the EGFR inhibitor Gefinitib (GEF), showing that Erk activation as indicated by phosho-Erk1/2 (pErk1/2) depends on EGFR activity. (e,f) actin was used as a loading control (g) BrdU incorporation assay showed no significant difference in cell proliferation between 2 biological replicates of Ctr and Ecad$^{-/-}$ keratinocytes with Kruskal-Wallis, Dunn's post hoc test (4 technical replicates each). Scale bars, 20µm. (h) Representative Western blot experiment for indicated proteins after transient transfection of GFP, full length E-cadherin (Ecad) or truncated E-cadherin lacking the β-catenin binding site (EcadΔβ) into E-cad$^{-/-}$ keratinocytes with Ctr as control. Right panel shows expression level of transfected E-cadherin constructs. (i). Quantification of western blot analysis for phospo-Erk (pErk) levels as indicator of EGFR signaling as represented in right panel (g). Dots represent biological replicates. *P=>0.05; n=5-6 biological replicates each group with one sample t-test, hypothetical value=1 (normalized to Ecad$^{-/-}$). (j) Western blot analysis for indicated proteins (related to Fig. 7g) showing phosphor EGFR in presence or absence of blebbistatin to inhibit actomyosin (72h in high Ca^{2+}, blebbistatin added after 24h).

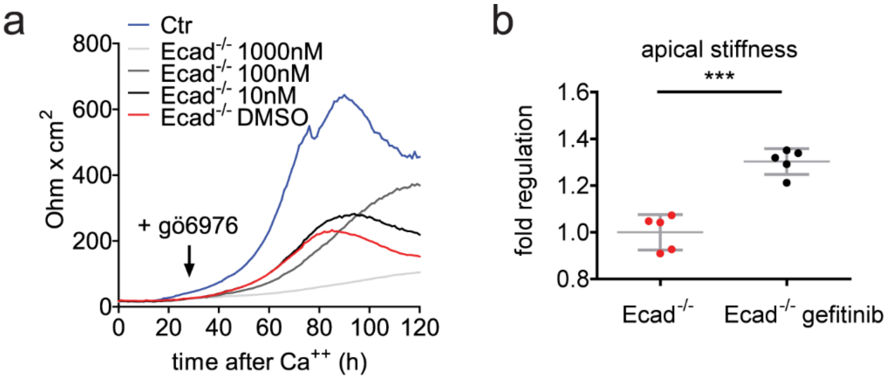

Supplementary Figure 8 E-cadherin regulates EGFR downstream signaling. (a) Transepithelial resistance (TER) measurements in primary keratinocytes in absence or presence of increasing doses of PKCα,β1 inhibitor Gö6976. (b) Quantification of relative difference in Young's moduli in DMSO and gefitinib treated E-cadherin$^{-/-}$ cells. Median values of biological replicates were used for statistical analysis. ***P<0.0001; E-cadherin$^{-/-}$/E-cadherin$^{-/-}$+gefitinib n=5 biological replicates with Student's t-test (>300 measurements per replicate).

Fig. 7e

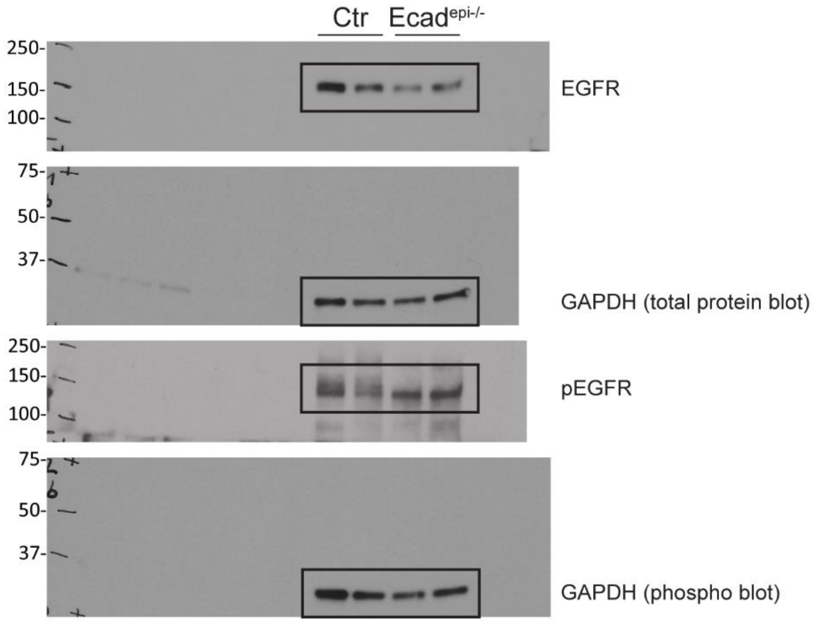

Supplementary Figure 9 Uncropped blots for Fig. 7e. The bands used in the figure are marked in black squares.

Supplementary Methods

Skin sample preparation

Paraffin and cryo sections were collected from the dorsal or ventral skin of newborn mice or embryonic stages as indicated. For paraffin sections, skin samples were spread on whatman paper and incubated in 4%PFA at 4°C over night. Tissue was kept in 70% EtOH prior to tissue infiltration via perfusion with Xylol and paraffin embedding. For cryo sections, skin samples were immediately embedded using Tissue-Tek O.C.T Compound (Sakura Finetek) on dry ice and fixed prior to staining. For vinculin stainings on embryonic skin, embryos were decapitated and fixed in 4% PFA over night at 4°C. Embryos were then consecutively incubated in 10%/20%/30% sucrose/PBS for 24h each concentration at 4°C. Paraffin and cryo blocks were sectioned for immunohistochemistry (5-7µm) and dried on Superfrost Plus microscope slides (Thermo Fisher Scientific).

Immunohistochemistry

For immunofluorescence on paraffin sections, sections were deparaffinized via two Xylene-washes 5min, followed by rehydration though an ethanol series (Xylene, Isopropanol, 100%, 75%,50%, 5min each) and two washes in H_2O. Slides were washed in PBS and transferred into Antigen retrieval buffer pH6 or pH9 (DAKO) depending on the used primary antibodies. Antigens were unmasked with the 2100 Retriever (Proteogenix) using high temperature and pressure. Cryo section were washed in PBS to remove OCT prior to fixation. Skin sections were encircled with a fat pen and blocked with 10% Normal goat serum (or Normal donkey serum) and 1% BSA in PBS for 1h at RT. Primary antibodies were diluted in ADS (antibody diluent solution, DAKO) and incubated overnight at 4°C in a humidified chamber. Sections were washed three times for 10min in PBS and incubated with the secondary antibodies and DAPI diluted 1:500 in ADS for 1h at RT in a dark humidified chamber. Secondary antibodies were washed off with PBS. Sections were mounted using Gelvatol (Calbiochem).

Protein Isolation and Immunoblotting

Epidermis was separated from dermis and dissociated with a MixerMill homogenizer and lysed in 1% SDS; 5mM EDTA lysis buffer. Cells were lysed on cell culture vessels by adding 95°C crude lysis buffer (1% SDS; 5mM EDTA) onto the cell culture plate. Lysates were scraped off the surface using cell scrapers. Cell and tissue suspension was homogenized by passing it through a 27-gauge syringe needle. Lysates were afterwards incubated for 5min at 95°C and stored at -20°C. Lysates were separated on SDS-PAGE gels (Novex), transferred to a PVDF membrane, blocked in either 5% western blot blocking solution (Roche) or 5% milk and incubated with primary antibodies followed by incubation with the appropriate horseradish peroxidase coupled secondary antibodies and detected using ECL (Thermo Fisher Scientific). Band intensities were quantified using Fiji.
