# Supplementary material for: E-cadherin integrates mechanotransduction and EGFR signaling to control junctional tissue polarization and tight junction positioning
Source: Nat Commun. 2017 Nov 1;8:1250. doi: 10.1038/s41467-017-01170-7 (PMC5665913; doi:10.1038/s41467-017-01170-7)
Supplement: Supplementary file 3 — Description of Additional Supplementary Files [file 41467_2017_1170_MOESM3_ESM.pdf]

Description of Additional Supplementary Files

File Name: Supplementary Movie 1
Description: **Epidermal tight junctions form at the apical end of a lateral vinculin positive adherens junction network in the granular layer 2.** Animation of the distribution of epidermal tight junction as indicated by ZO-1 (purple) and tension-bearing adherens junction as indicated by vinculin (green) recruitment. The movie is derived from a confocal stack projection of the granular layer 2 from a newborn mouse epidermal whole mount staining.

File Name: Supplementary Movie 2
Description: **F-actin is polarized across epidermal layers with scattered F-actin^{high} cells in the SG1 layer.** Animation of the distribution of F-actin across the epidermal layers, showing concentrated cortical F-actin in cells throughout the granular layer 2 and even more F-actin concentration in scattered cells of the granular layer 1. The movie shows a full thickness confocal stack projection from a newborn mouse epidermal whole mount staining.
